# Ecological and evolutionary factors of intraspecific variation in inducible defenses: Insights gained from *Daphnia* experiments

**DOI:** 10.1002/ece3.6599

**Published:** 2020-08-03

**Authors:** Mariko Nagano, Hideyuki Doi

**Affiliations:** ^1^ Graduate School of Simulation Studies University of Hyogo Kobe Japan

**Keywords:** *Daphnia*, inducible defense, intraspecific variation, kairomone, phenotypic plasticity

## Abstract

Phenotypic variation among individuals and species is a fundamental principle of natural selection. In this review, we focus on numerous experiments involving the model species *Daphnia* (Crustacea) and categorize the factors, especially secondary ones, affecting intraspecific variations in inducible defense. Primary factors, such as predator type and density, determine the degree to which inducible defense expresses and increases or decreases. Secondary factors, on the other hand, act together with primary factors to inducible defense or without primary factors on inducible defense. The secondary factors increase intraspecies variation in inducible defense, and thus, the level of adaptation of organisms varies within species. Future research will explore the potential for new secondary factors, as well as the relative importance between factors needs to be clarified.

## Graphical abstract

We identified seven secondary factors causing variations in inducible defense based on previous studies: abiotic factors, ecological and evolutionary traps, food, alarm substance, clone/genotypes, instars, and maternal effect.

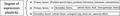

## 1 INTRODUCTION

Organisms can change their phenotypic traits (morphology, behavior, and physiology) and adapt to environmental variations. The ability of a single genome to produce a range of phenotypes in response to environmental conditions is called phenotypic plasticity (Agrawal, 2001; Fordyce, 2006). In general, the degree of phenotypic plasticity has a direct effect on fitness and therefore represents an important feature of the organism's adaptation.

The change in traits observed in phenotypic plasticity may not be binary (high and low) or represented by an on/off reaction but rather a continuous process in individuals (Auld, Agrawal, & Relyea, 2010; Forsman, 2015). Owing to this variation, individual organisms differ in cost and/or adaptive status relative to that of the optimal phenotype in a giving environment. Costs of inducible phenotypes are a central component of the evolution of plasticity (Auld et al., 2010; DeWitt, Sih, & Wilson, 1998) but have proven difficult to measure empirically. Variation in phenotypic plasticity can produce several adaptive states (i.e., adaptive, maladaptive, or neutral); therefore, studies of phenotypic plasticity tend to focus on cost detection and adaptation status (Auld et al., 2010; Murren et al., 2015). Because even trait variation of phenotypic plasticity is linked to evolution (Bolnick et al., 2011), it is important to clarify why variance in plasticity traits occurs and is maintained in the environment.

Predation is an important factor driving natural selection, and defensive traits are expressed against predators in a plastic or constitutive manner. *Daphnia* (Arthropoda Crustacea) is an excellent model system for studying predator‐induced plasticity (Lass & Spaak, 2003; Tollrian & Dodson, 1999), with alterations in their phenotype against predators including changes in body size, head shape, tail length, number of eggs, reproduction status, and distribution depth (Lass & Spaak, 2003). To express predator‐induced plasticity, *Daphnia* need to perceive predatory kairomone (chemical substance) and/or other factors besides predators; the former is called primary factor and the latter secondary factor (Riessen & Gilbert, 2019). Riessen and Gilbert (2019) suggested in a review that secondary factors are related to increases or decreases in the degree of plasticity. This suggests that predator‐induced plasticity displays different trait values among individuals owing to the interaction between primary and secondary factors. Therefore, a wide range of factors can induce predator‐induced plasticity. Considering variations in predator‐induced plasticity, it is important to consider how secondary factors as well as the essential triggers work. There are numerous studies focusing on the predator‐induced plasticity of *Daphnia*, making it potentially feasible to target and synthesize the various secondary factors affecting variations in this plasticity. *Daphnia* are tractable in various experimental settings and can be analyzed with modern genomic tools (Miner, De Meester, Pfrender, Lampert, & Hairston, 2012) and large‐scale gene expression technology (Colbourne et al., 2011). Specifically, *Daphnia pulex* is the first crustacean to have its whole genome sequenced (Colbourne et al., 2011). Moreover, multiple studies of *Daphnia* have identified the neural mechanisms associated with predator‐induced defenses (Miyakawa, Sugimoto, Kohyama, Iguchi, & Miura, 2015; Weiss & Tollrian, 2018). It can also be argued that, based on the predator–prey system, the elucidate secondary factors regulating variations in *Daphnia* plasticity could lead to a deeper understanding of phenotypic plasticity.

The goal of this review is to clarify variations in predator‐induced plasticity in *Daphnia* and summarize the secondary factors influencing those variations. We begin with a brief overview of variations of inducible defenses in *Daphnia* and then examine the relationship between plasticity variation and the various secondary factors involved. Recent theoretical works indicate that intraspecific trait (nonplasticity) variation can have significant ecological effect (Bolnick et al., 2011) and the variation of degree of expression in inducible defense might have likewise significant relationship ecological and evolutionary context. Exploring such variations associated with inducible defense is a critical step in clarifying how changes in traits occur and are maintained according to the environment.

## 2 REVISITING THE IMPORTANCE OF VARIATIONS IN INDUCIBLE DEFENSE

Ecologist have long recognized intraspecific variation in inducible defense; here we explore the factors involved in intraspecific variation in the inducible defense of *Daphnia* and synthesize the findings reported by empirical studies. Phenotypic changes show both qualitative (the presence or absence of spines) and quantitative (body size, spine length, and/or migration behavior) traits. Moreover, *Daphnia* express a combination of several unique, species‐specific defensive traits in response to chemical cues (self‐induced defense; a primary factor) initiated by predators, such as fish and invertebrates (Boeing, Ramcharan, & Riessen, 2006a, 2006b; Boersma, Spaak, & De Meester, 1998). Although predator‐induced plasticity in *Daphnia* includes a broad range of traits and shows complicated expression patterns, studies might underestimate or overestimate the variation based on evaluation of only average values for a single trait. Stoks, Govaert, Pauwels, Jansen, and De Meester (2016) used univariate and multivariate analyses of phenotypic plasticity to identify a natural *Daphnia magna* population capable of rapidly tracking changes in fish predation. This integrated, multi‐trait approach improved our understanding of the evolution of phenotypic plasticity. The combined value of all the variation capacities of an individual (growth stage and multiple traits) in phenotypic plasticity would be measured as a potential capacity for adaptation.

Even if a change in one trait appears to be adaptive, other traits may appear to be maladaptive. This discrepancy is referred to as “trait compensation” (DeWitt, Sih, & Hucko, 1999) and suggests that the adaptability of an individual cannot be measured using only one trait. Specific traits complement one another, and inducible defenses can show both progression and regression of multiple traits in an individual (Boeing et al., 2006b; Boersma et al., 1998). In fact, these can occur simultaneously, which warrants the simultaneous observation of multiple traits. From a cost‐benefit perspective, *Daphnia* might develop only a few inducible defense characteristics (Boersma et al., 1998), indicating that the expression of multiple defensive traits is associated with a certain cost in the forms of maintenance, production, and information acquisition. If a single trait is sufficient as an inducible defense against multiple predators, it could be unnecessary to develop multiple defensive traits. For example, development of only an elongated spine can make it more difficult for *Daphnia* to be captured by several predators (Caramujo & Boavida, 2000), which lowers the cost of acquiring this characteristic (Laforsch & Tollrian, 2004b). In this situation, the costs remain the same, but the benefits increase if it helps against multiple predators at once.

The primary factor is the most important aspect of variation in inducible defense in *Daphnia*. The factors of predators can be separated into “predator species/type,” “predatory kairomone,” and “kairomone concentration” as main or primary factors. First, *Daphnia* must contend with predators that are size‐selective regarding to their prey (Dodson, 1974). The predation type for invertebrates is generally gape‐limited predation that shows preference for small zooplankters, whereas vertebrate predators, such as fish, tend to be large zooplankters (Brooks & Dodson, 1965). Therefore, *Daphnia* will know exactly what kinds of predators are existing there and will express a moderate degree of defense accordingly. In a meta‐analysis, Riessen (1999) showed that the life history responses of *Daphnia* to *Chaoborus* larvae differ substantially from those to *Notonecta* and fish. In the presence of small‐size‐selective predation by *Chaoborus* larvae, *Daphnia* mature later and show a larger size at that time. By contrast, under large‐size‐selective predation by fish, *Daphnia* reproduce early and are small at maturity (Riessen, 1999). *Daphnia* sizes vary among species (Gliwicz, 1990); body size is an important factor in terms of inducible defense traits.

The essential trigger includes *predatory kairomone* or *kairomone concentration*. Several studies report strong evidence for dose dependence where inducible defense is concerned (Dennis, Carter, Hentley, & Beckerman, 2010; Hammill, Rogers, & Beckerman, 2008; Parejko & Dodson, 1990), and the degree of defense expression tends to vary directly with predator abundance or kairomone concentration. However, studies show that the degree of dose‐specific plasticity does not increase indefinitely as kairomone concentration increases, but reach a saturation point beyond which no additional changes in plasticity occur (Hammill et al., 2008; Reede, 1995; Weetman & Atkinson, 2002). This suggests that plasticity expression is constrained by what is not predatory kairomone.

## 3 CATEGORIZED FACTORS ASSOCIATED WITH VARIATIONS IN INDUCIBLE DEFENSE

**FIGURE 1:**
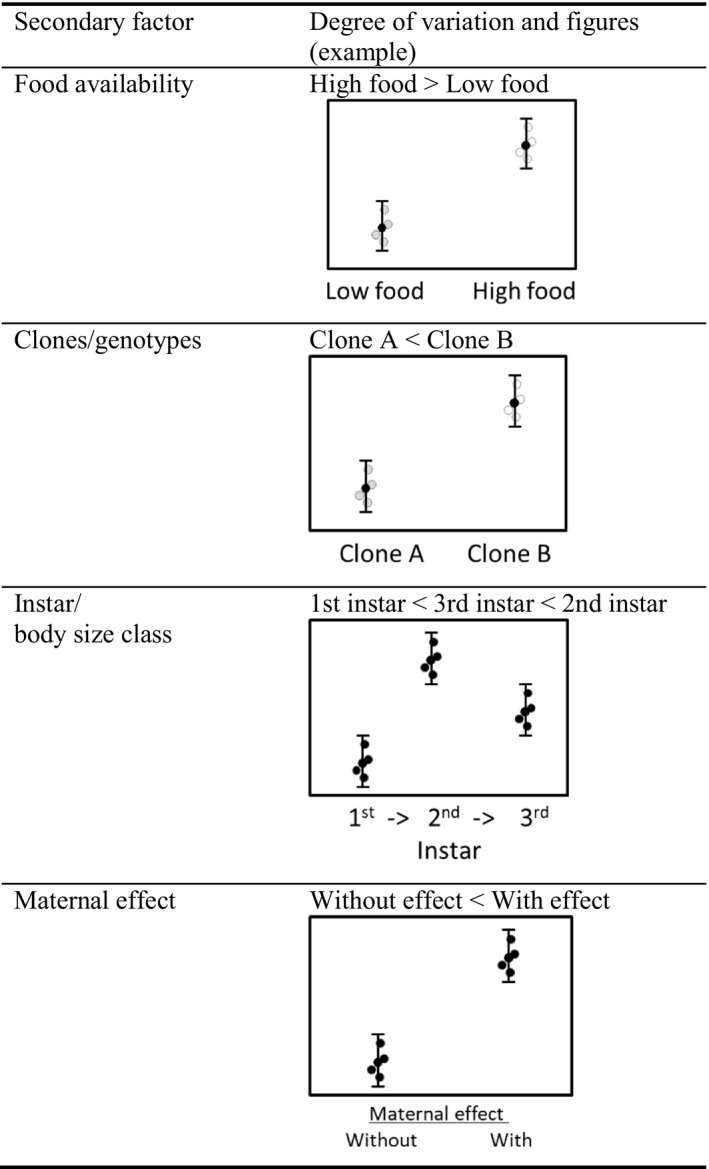
Classification of secondary factors affecting the degree of defense

We identified seven secondary factors causing variations in inducible defense based on previous studies (Figure 1): abiotic factors, ecological and evolutionary traps, food, alarm substance, clone/genotypes, instars, and maternal effect. The following three factors were not noted owing to the paucity of prior research or controversy: abiotic factor, ecological trap, and alarm cue (Figure 1).

**FIGURE 2:**
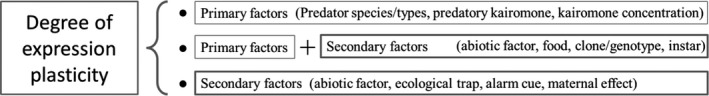
Conceptual diagram outlining the factors of intraspecific variations in predator‐induced plasticity

The seven factors can be distinguished by their relative relationship to primary factors (Figure 2). One is the primary factors to promote or inhibit the degree of expression in inducible defense by working with the primary factors; abiotic factors, food, clone/genotype, and instars. The other is the secondary factor alone can express predator‐induced plasticity, but the degree of expression may be equivalent, smaller, or larger compared with the induction traits from the primary factor: abiotic factors, ecological evolutional traps, alarm substance, and maternal effect. If organisms can express an inducible defense with as few factors as possible, it would be adaptive to take less cost than to perceive a number of factors. To the cost of factor acquisition (DeWitt et al., 1998), organisms would try to assess environment to express phenotype‐environment matching. Given the avoidance of mismatching phenotypes, secondary factor may help the control, accelerate, and limit of the expression of defensive plasticity, in addition to ensuring the reliability of primary factors.

### 3.1 Abiotic factors

Organisms may remember more accurate and reliable cues in order to predict and to know the presence of predators, although reliable cue selection mechanisms are unknown. If the emergence of predators is seasonal/temporal, *Daphnia* may be able to detect and respond to abiotic seasonal factors. Abiotic factors, including water temperature (Bernot, Dodds, Quist, & Guy, 2008; Hanazato, 1991; Lass & Spaak, 2003; Sakwinska, 1998; Weetman & Atkinson, 2002; Yurista, 2000), turbulence ( Laforsch & Tollrian, 2004b; 2006), light (Boeing, Leech, Williamson, Cooke, & Torres,^2004^ ; Rhode, Pawlowski, & Tollrian, 2001; Rose, Williamson, Fischer, Connelly, Olson, & Noe, 2012 ), and copper and other minerals (Hunter & Pyle,^2004^ ; Mirza & Pyle, 2009), can affect the degree of predator‐induced plasticity, but there is no fixed trend. These factors may work together with the primary factors, or they may work on their own. These abiotic factors may change the chemical composition of the predatory kairomone and thus reduce their effect on the organism. Temperature manipulation has shown that the degree of plasticity varies with differences in temperature alone, regardless of kairomone concentration (Sakwinska, 1998), and that other crustaceans have spines that elongate in the absence of kairomone but only at high temperatures (Miehles, McAdam, Bourdeau, & Peacor, 2013). Since these abiotic factors strongly influence the survival and life history traits of daphniids in the first place, abiotic factors may often limit expression plasticity even when the primary factors are detected.

The degree of expressed plasticity is thought to be both enhanced and suppressed in such environments and may be enhanced when *Daphnia* links periodic changes (i.e., seasons) in predator presence to physical stimuli and may be suppressed in the absence of relationships with cycles (Riessen & Gilbert, 2019). Miehles and her colleagues, studying the plasticity of *Bythotrephes*, have called this type of factor a “proxy cue” (Miehles et al., 2013). These factors are associated with local predator regimes and thereby cause intraspecific variation between populations. If primary factors are not reliable cues of predation risk, the abiotic factors would be accurate and useful factors. Moreover, abiotic factors that correlate with selective agents work similarly to primary factors and alone can cause an inducible defense on their own (Miehles et al., 2013). The phenomenon of inducible defense without primary factors is well known, although there is a lack of experimental support for identifying these factors. This factor may be the most reliable cue of the emergence, presence, and predation cycle of predators that is closest to *Daphnia* itself.

### 3.2 Ecological and evolutionary traps

Organisms can incorrectly express phenotypes owing to artificial changes in the environment (i.e., an “ecological trap”; Schlaepfer, Runge, & Sherman, 2002), and the expression of inducible defenses can be affected by artificial cues (i.e., abiotic cues, as noted here). Even in the absence of predators, *Daphnia* can be triggered by anthropogenic chemicals (xenobiotics), such as pesticides (Crispo et al., 2010). For example, *Daphnia retrocurva* in urban lakes display defensive vertical migration in the presence of a predator and use bright light as a cue (Moore, Pierce, Walsh, Kvalvik, & Lim, 1998). Although this was not interpreted in the context of an ecological trap, it was suggested that incorrect inducible defenses became maladaptive. Intraspecific variation caused by ecological traps within populations can become maladaptive; therefore, it is necessary to understand the degree of variation and the evolution of maladaptation.

### 3.3 Food

Food level is not only a basic element of growth, but also a critical factor in modifying inducible defenses (e.g., depth‐selective behavior [Loose & Dawidowicz, 1994]; morphological defenses [Tollrian,1995b] 1995a, 1995b; life history traits [Jeyasingh & Weider, 2005; Stibor & Navarra, 2000; Weetman & Atkinson, 2002]). For instance, inducible defense under low food level is expressed, but to a lesser extent (Barry, 1995). The degree of expressed plasticity has been found to be greater at high food levels and lower at low food levels, with other clones responding in the opposite direction (Jeyasingh & Weider, 2005). In predation experiments on the same size *Daphnia* raised under different food conditions, *Daphnia* clones under low food conditions were more likely to be preyed by *Chaoborus* larvae easy (Jeyasingh & Weider, 2005). However, it is worth nothing that *Daphnia* clones in the high food condition had a more variable susceptibility to be eaten. The rich food conditions may give *Daphnia* a variety of ways to adaptation.

### 3.4 Alarm substances

Alarm substances from crushed conspecifics act as enhancers of change (Laforsch, Beccara, & Tollrian, 2006; Pijanowska, 1997 ; Pijanowska & Kowalczewsk, 1997; Stabell, Ogbebo, & Primicerio, 2003); however, there are also reports indicating almost no change caused by alarm substances (Parejko & Dodson, 1990; Stirling, 1995; Walls & Ketola, 1989). Given intraspecific variation, both results are possible. Alarm cues may not be sufficient to identify species predators, and the set of defensive traits subsequently expressed may be misleading, but it does provide reliable evidence of being captured during the predation cycle. Unless it is a specific defense against specific predator, express an inducible defense by this cue may be adaptive. This alarm cue is thought to spread across a narrow range, resulting in variations in plasticity between individuals according to their receipt of the cue. Without widespread diffusion of alarm cues, individuals would not experience the same concentration of cues, and hence, there would be differences in how they react.

### 3.5 Clones/genotypes

The degree of expression plasticity commonly varies between clones (morphological defense, Boeing et al., 2006; Declerck & Weber, 2003; Ferrari, Müller, Karaaijeveld, & Godfray, 2001; Havel, 1985; Hammill et al., 2008; Jeyasingh & Weider, 2005; Lively, Hazel, Schellenberger, & Michelson, 2000; Miyakawa et al., 2015; Rabus & Laforsch, 2011; Spitze, 1992; Weider, 1985; Wiąckowski, Fyda, Pajdak‐Stós, & Adamus, 2003, life history traits ; Weider & Pijanowska, 1993, and behavioral traits Michels, Amsinck, Jeppesen, & De Meester, 2007). Interclonal variation in the expression of inducible defenses originates from habitats with different predation regimes (Boeing et al., 2006a; Boersma et al., 1998, 1999; Dennis et al., 2010). The interclonal variations in the type and degree of inducible defense of *Daphnia hyalina* result from seasonal variations in the clonal composition of field populations (1985 Stibor & Lampert, 2000). Moreover, this might partly account for the seasonally different occurrence of defended and undefended morphs in the field, caused by changing predator regimes (Havel, 1985). The variation in degree of expression inducible defense is predicted might be greater between species than between clones, although no comparisons have been made. However, clonal variations are not negligible or small enough to be ignored. If the variation in the degree of plasticity is greater for clonal variation than for interspecific variation, then natural selection might be working strongly within the species.

### 3.6 Instars

Although it is unclear how *Daphnia* itself perceives own body size, the body size is an important factor in determining the extent to which inducible defense should be expressed (Hart & Bychek, 2011; Tollrian, 1995a, 1995b). This is because predation sensitivity changes with age/instar changes in body size. It is important to be able to identify the type of predator, that is, gape‐limited or visual predator, by primary factors at first. *Chaoborus* larvae prefer a narrow range of small‐sized prey (Pastorok, 1981; Swift & Fedorenko, 1975), whereas fish prefer larger‐sized prey, because they are readily visible (Brooks & Dodson, 1965; Nunn, Tewson, & Cowx, 2012). Hence, inducible defense varies among instars. For example, neckteeth induction is stronger at the 2nd and 3rd instars of *Daphnia* than at other stages (Tollrian, 1993; Tollrian, 1995a, 1995b; Imai, Naraki, Tochinai, & Miura, 2009), because the former are the most vulnerable to *Chaoborus* larva predation. Therefore, depending on the trait, the degree of expression plasticity can be varied large within instar. The presence of fish chemicals decreases *Daphnia* body size (Boersma et al.,1999  Brett, 1992; Carter, Silva‐Flores, Oyanedel, & Ramos‐Jiliberto, 2013; Fisk, Latta, Knapp, & Pfrender, 2007; Weber & Declerck, 1997). *Daphnia* expresses inducible defense throughout its entire lifespan in the presence of predators capable of ingesting prey of any size (Laforsch & Tollrian, 2004b; Rabus & Laforsch, 2011).

### 3.7 Maternal effect

Inducible defense can be transmitted to the next generation as a history of predation. The degree of defensive traits in the daughter generation of *Daphnia cucullata* depends on the extent to which the maternal line was exposed to predation by *Chaoborus* larvae (Agrawal, Laforsch, & Tollrian, 1999). The exposure of kairomone during embryonic and postembryonic development of *D. pulex* is required to allow adequate extension of head length.(Dennis, LeBlanc, & Beckerman, 2014; Miyakawa et al., 2010). However, not all plasticity traits are dependent on maternal effects (Mikulski & Pijanowska, 2017), and it is adaptive because the next generation can express the defensive trait without the cost of perceiving primary factors.

## 4 CONCLUSION

The variation of degree in inducible defense of *Daphnia* among conspecific individuals has long been recognized in experimental and field work. Despite a fast‐growing study on the variation in inducible defense, we lack a general framework for understanding the variation by which factors influences to express. Then, we classified seven secondary factors related to evolutionary and ecology in predator‐induced plasticity. The secondary factors can be distinguished by their relative relationship to primary factors, that is, presence of predator and/or predatory kairomone. Abiotic factors, food, clone/genotype, and instars are promoted or inhibited the degree of expression in inducible defense by working with primary factors. And while abiotic factors, ecological traps and alarm substance, and maternal effect may work alone, but the degree of expression by them may be equivalent, smaller, or larger compared with the degree of variation from the primary factors. Variation of inducible defense is associated with vulnerability of predator. Therefore, it will be important to clarify the factors and the degree of variation in the future.

## 5 FUTURE DIRECTIONS

Research into inducible defenses in field populations is informative; however, recent studies were often based on laboratory experiments. In the laboratory, predatory kairomones are prepared based on a “kairomone recipe” that is generally established at a much higher concentration than that in nature. It is believed that *Daphnia* will react sufficiently in the presence of appropriate stimuli; therefore, preparation of a “kairomone recipe” does not assume the same response in any population of any species. Additionally, the　degree of expression of inducible defense inducible defenses differ among populations of the same species owing to local adaptation (Boersma, De Meester, & Spaak, 1999; Boeing et al., 2006a; Reger, Lind, Robinson, & Beckerman, 2018). Therefore, experiments might overestimate or underestimate intraspecific variations. It is necessary to investigate dose–response curves based on initial changes in predator density, because the “kairomone recipe” already sufficiently induces defensive traits. Inducible defense experiments can be constructed using chemical substances based on a given predator, because the chemical compositions of the *Chaoborus* (Weiss et al., 2018) and fish kairomones have been identified. And experimental individuals are maintained in a simpler environment than that which occurs in natural habitats. *Daphnia* may be used to analyze the genetic background of clones in order to elucidate how plasticity expression during a lifetime varies among factors. The relationship between traits and genetic analysis of the clones should be validated with laboratory experiments, long‐term field studies.

There remain other unresolved issues. For example, one phenomenon not yet elucidated is extraordinary inducible defenses reported by field observations (Laforsch & Tollrian, 2004a; Tollrian & Laforsch, 2006). Such defenses developed by *Daphnia* have not been successfully reproduced in the laboratory, likely because plasticity is expressed by a plurality of secondary factors. The degree of plasticity in *Daphnia* according to field observation can be high except when predator density is high (Luecke & Litt, 1987; Nagano & Doi, 2018). We will attempt to elucidate the reasons for the discrepancy between experimental and field specimens in terms of their comparative degrees of inducible defense expression.

A major goal of evolutionary biology is to understand the mechanisms involved in creating biodiversity. Recent data concerning variations in phenotypic plasticity have promoted ecological speciation but with little empirical evidence (Pfennig et al., 2010). Although speciation involves several processes (Pfennig et al., 2010), phenotypic plasticity is thought to be helpful in the early stages of speciation (Forsman, 2015; Pfennig et al., 2010; Snell‐Rood, 2013; Thibert‐Plante & Hendry, 2011). As the most famous example, tadpoles of *Spea multiplicate* may facilitate speciation based on resource‐induced plasticity in omnivorous or carnivorous morphology depending on resource availability (Pfennig & McGee, 2010). In this case, both morphologies eventually separate by intraspecific variations in plasticity. This example shows that during the onset of speciation for resource utilization, spatiotemporal distribution remains the same, whereas there is variation in morphology. Similar to resource‐induced plasticity, phenotypic plasticity against predation (inducible defense) creates morphological variance. Unfortunately, high‐quality empirical data do not yet exist for speciation of *Daphnia*. However, a variety of factors can cause intraspecific variation in *Daphnia* plasticity of inducible defense, and few experimental studies discuss how this intraspecific variation is maintained or how it is linked (or not linked) to speciation. We believe that these factors and variations will provide information regarding their effect on the early stages of speciation. Fortunately, *Daphnia* is useful for these kinds of experiments owing to its short generation time, ease of breeding, and the capability of using dormant eggs from previous generations. Future studies should focus on tracking both traits and genotypes through long‐term evolution experiments in order to reveal how various traits that appear disadvantageous are conserved.

Because water temperature is a major secondary factor, research into the phenotypic plasticity of living organisms in response to climate change will become increasingly significant in the future (Crispo et al., 2010; Weiss et al., 2018). Future studies should still consider not only the response of physiological activity against climate change, but the effect on predator–prey dynamics.

Animal personality remains constant, regardless of environmental variation (Dingemanse et al., 2009; Sih, Bell, Johnson, & Ziemba, 2004; Wolf & Weissing, 2012), and inducible defenses can vary because of personality differences (e.g., bold and shy) regardless of the presence of predators (crucian carp; Hulthén, Chapman, Nilsson, Hollander, & Brönmark, 2014). This study showed that bold individuals undergo more substantial morphological changes than shy individuals. In contrast, shy individuals vary considerably in terms of evasion behavior. Therefore, personality‐induced variation in inducible defense may be seen as both an adaptive and a maladaptive response. Under various environments and situations within the same species, bold individuals will have wide activity ranges, whereas shy individuals will have a narrow range. As *Daphnia* seem to have a personality (Heuschele, Ekvall, Bianco, Hylander, & Hansson, 2017), this species merits further investigation of personality as a factor contributing to variations in inducible defense. Depending on personality, the degree of expression in plasticity is expected to vary, as in the case of the crucian carp.

## CONFLICT OF INTEREST

The authors declare no conflicts of interest.

## AUTHOR CONTRIBUTION

**Mariko Nagano:** Conceptualization (lead); Writing‐original draft (lead). **Hideyuki Doi:** Validation (lead).

## Data Availability

Data sharing not applicable to this article as no datasets were generated or analyzed during the current study.
